# Investigating the feasibility of motorcycle autonomous emergency braking (MAEB): Design criteria for new experiments to field test automatic braking

**DOI:** 10.1016/j.mex.2021.101225

**Published:** 2021-01-10

**Authors:** Cosimo Lucci, Mirko Marra, Pedro Huertas-Leyva, Niccolò Baldanzini, Giovanni Savino

**Affiliations:** a Department of Industrial Engineering, University of Florence, Via di Santa Marta 3, 50139 Florence, Italy; b Monash University Accident Research Centre, 21 Alliance Ln, Clayton, VIC 3800, Australia

**Keywords:** Motorcycle, Active safety, Autonomous emergency braking, Field testing, Test protocol

## Abstract

Autonomous Emergency Braking (AEB) was proved to be an effective and reliable technology in reducing serious consequences of road vehicles crashes. However, the feasibility in terms of end-users’ acceptability for the AEB for motorcycles (MAEB) still has to be evaluated. So far, only Automatic Braking (AB) activations in straight-line motion and decelerations up to 2 m/s^2^ were tested with common riders.

This paper presents a procedure which provides comprehensive support for the design of new experiments to further investigate the feasibility of MAEB among end-users. Additionally, this method can be used as a reference for designing tests for other advanced rider assistance systems.

• A comprehensive literature review was carried out to investigate previous findings related to MAEB. After that, a series of pilot tests using an automatic braking device on an instrumented motorcycle were performed.
• The specifications for new AB experiments were defined (in terms of test conditions, participants requirements, safety measures, test vehicles and instrumentation).
• A test protocol was defined to test the system in different riding conditions and with different AB working parameters. A proposal for the data analysis was presented.

## Graphical abstract

**Figure:**
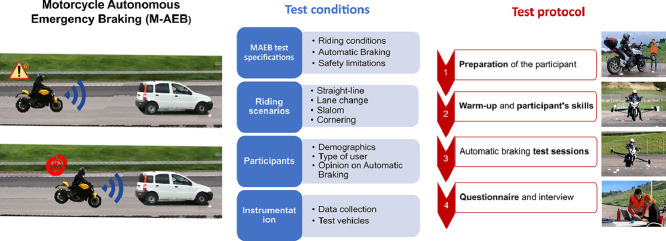

Specifications table

**Table:** 

Subject Area:	Engineering
More specific subject area:	*Road safety*
Method name:	Field test autonomous emergency braking for powered-two-wheelers with participants
Name and reference of original method:	*The method proposed in this paper is an advancement of test protocol proposed in a previous study: G. Savino, M. Pierini, J. Thompson, M. Fitzharris, M.G. Lenné, Exploratory field trial of motorcycle autonomous emergency braking (MAEB): Considerations on the acceptability of unexpected automatic decelerations, Traffic Inj. Prev. 17 (2016) 1–12*. https://doi.org/10.1080/15389588.2016.1155210.
Resource availability:	*N.A.*

**Method details**

## Method goal

To extend the tests to new conditions that are relevant for Motorcycle Autonomous Emergency Braking (MAEB), especially for the vehicle dynamics, while granting safety of participants and a good approximation of real-world conditions, to test the acceptability of new deceleration values.

## Test methods

The guidelines and the test protocol presented in the following sections were developed through a process of literature review and pilot testing (see the section “Process employed to define the proposed test method” in the “Additional information” of this paper).

For this study, all subjects gave their informed consent for inclusion before they participated. The study was conducted in accordance with the Declaration of Helsinki, and the protocol was approved by the Ethics Committee of the University of Florence (Decision N. 46, 20/03/2019).

### MAEB test specifications

In order to field-test the Motorcycle Autonomous Emergency Braking system, in previous studies [1], [2], [3] Automatic Braking (AB) events were studied. Participants were exposed to an automatic deceleration through an automatic braking action executed by the motorcycle without a braking action of the rider. Therefore, the first step for field-testing MAEB interventions with participants, is the definition of the riding conditions to be reproduced and the necessary AB functions and performance. Subsequently, the test protocol can be developed observing the safety limitations required to safeguard participants involved in the tests.

#### Riding conditions

As found in the literature, the intervention of MAEB was tested as unexpected activation without an obstacle [2,3], in order to reproduce a false activation or an intervention that gets unprepared the rider. This is indeed one of the most challenging working condition related to safe controllability of the vehicle and rider acceptability of the system. Therefore, the test protocol is arranged to make AB interventions unexpected for the rider, triggering AB pseudo-randomly without the presence of obstacles. To reduce the predictability, the interventions are not excessively repetitive and frequent (i.e. they are spread in time and in different spots along the test track; as reference value is one AB intervention every 100 s of riding). The overall number of AB intervention is as limited as possible, in order to reduce the learning effect, while ensuring a repetition of the assessment: for each maneuver tested and level of deceleration, the number of repetitions is very low (2–3). These values derived from the trade-off between different opposing requirements: the maximum duration of the trial and the number of different activation conditions.

The AB is deployed in a set of representative conditions for real-world applications, including also conditions where MAEB could be less efficient or even present issues. According to the results of previous studies, such conditions are common in an urban environment, characterized by low velocities (up to 50 km/h) and mixed maneuvers (e.g. short straight lines, curves, lane changes and traffic filtering). Since MAEB is a pre-crash braking system, which could be triggered less than one 1 s before the impact [4], the AB is tested for a similar duration without stopping the vehicle.

#### Automatic braking

According to the literature, the deceleration profile so-called “Block profile”, which is a constant deceleration time achieved through a constant jerk, is the best one among the few profiles tested so far [3]. This simple profile combines good acceptance by riders and easiness of implementation. In order to maximize the efficacy of MAEB, the constant fade-in jerk is as high as possible, but always under levels previously showed as significant thresholds for the controllability of Powered Two-Wheelers (PTWs) (−25 m/s^3^) [5]. At the end of the constant deceleration, a fade-out ramp with constant jerk is added to conclude the intervention; again, a maximum jerk of 25 m/s^3^ is adopted to avoid risks of destabilization and possible high-side events when the lateral dynamic is involved. The AB device is also provided with safety controls, in order to guarantee the highest level of safety to the participants. The safety controls include limitations to the conditions for the intervention of AB (e.g. in terms of speed of the vehicle, deceleration and roll angle) and a latency time (e.g. 5 s) between two consecutive activations to avoid multiple interventions.

#### Safety limitations

During the execution of the tests, the investigators observe the participant and monitor the appropriateness of the riding style and conditions before the activation of AB. If the test protocol consists of testing the intervention of the system in lateral maneuvers, such as curve, the test vehicle needs to be provided with outriggers, to prevent participants from low side fall. Nevertheless, AB field testing is related with residual risks (e.g.: person struck by test vehicle, fall from the vehicle due to loss of control, vehicle collision with obstacles, failure of the AB system, failure of test vehicle).

### Scenarios and maneuvers

A crucial point in the design of the field test is the definition of the test track employed to simulate an urban scenario. The track is intended to reproduce riding situations in which the speed and the performed maneuvers are similar to those observed in accidents in urban areas [6,7] and, more generally, in urban riding situations relevant for MAEB activation. Previous studies have focused on modeling the behavior of motorcycles in the urban scenario [8]. Aiming to reproduce this behavior on a test track, it is possible to simulate urban riding through four major maneuvers: straight-line riding, lane change, cornering and a slalom reproducing a complex vehicle dynamic condition such as traffic filtering.

#### Straight-line section

The sector of the test track emulating straight-line riding is constructed with a straight stretch suitably sized. The length of this section is chosen to allow the speed range typical of urban scenario (40–50 km/h) to be achieved (see Table 1). This section is sufficiently extended to obtain the first zone of acceleration and a second zone at constant speed where the AB is triggered. This is the maneuver where MAEB intervention could be easily applicable since AEB showed good benefits in rear-end and head-on crash scenarios [9], and it is the most tested maneuver in previous field studies [1], [2], [3].

**Table 1:** Maneuvers descriptive parameters.

Maneuver	Descriptive parameters	Notes
**Straight**	**Geometric parameters:** Straight-line minimum length: 80 m	
	**Kinematic parameters:** Speed range: 30–60 km/h Roll range: < +/- 5°	
**Lane change**	**Geometric parameters:** Obstacle width: 1.8 m Cross-lane section: 7 m Lanes width: 3 m	Reference [11]
	**Kinematic parameters:** Speed range: 30–50 km/h Roll range: +/- 15–25° Roll rate: 30–40°/s Yaw rate: 25–30°/s	Max value of roll to be reached during the maneuver Max value of roll rate to be reached during the maneuver Max value of yaw rate to be reached during the maneuver
**Slalom**	**Geometric parameters:** Number of markers 4 Markers distance 7 m	Reference [10]
	**Kinematic parameters:** Speed range: 25–40 km/h Roll range: +/- 15–25° Roll rate: 30–40°/s Yaw rate: 30–40°/s	Max value of roll to be reached during the maneuver Max value of roll rate to be reached during the maneuver Max value of yaw rate to be reached during the maneuver
**Curve**	**Geometric parameters:** Curve radius: 15–20 m	Defined by speed and maximum roll
	**Kinematic parameters:** Speed range: 25–40 km/h Roll range: 20–30°	

#### Lane change section

**Fig. 1:**
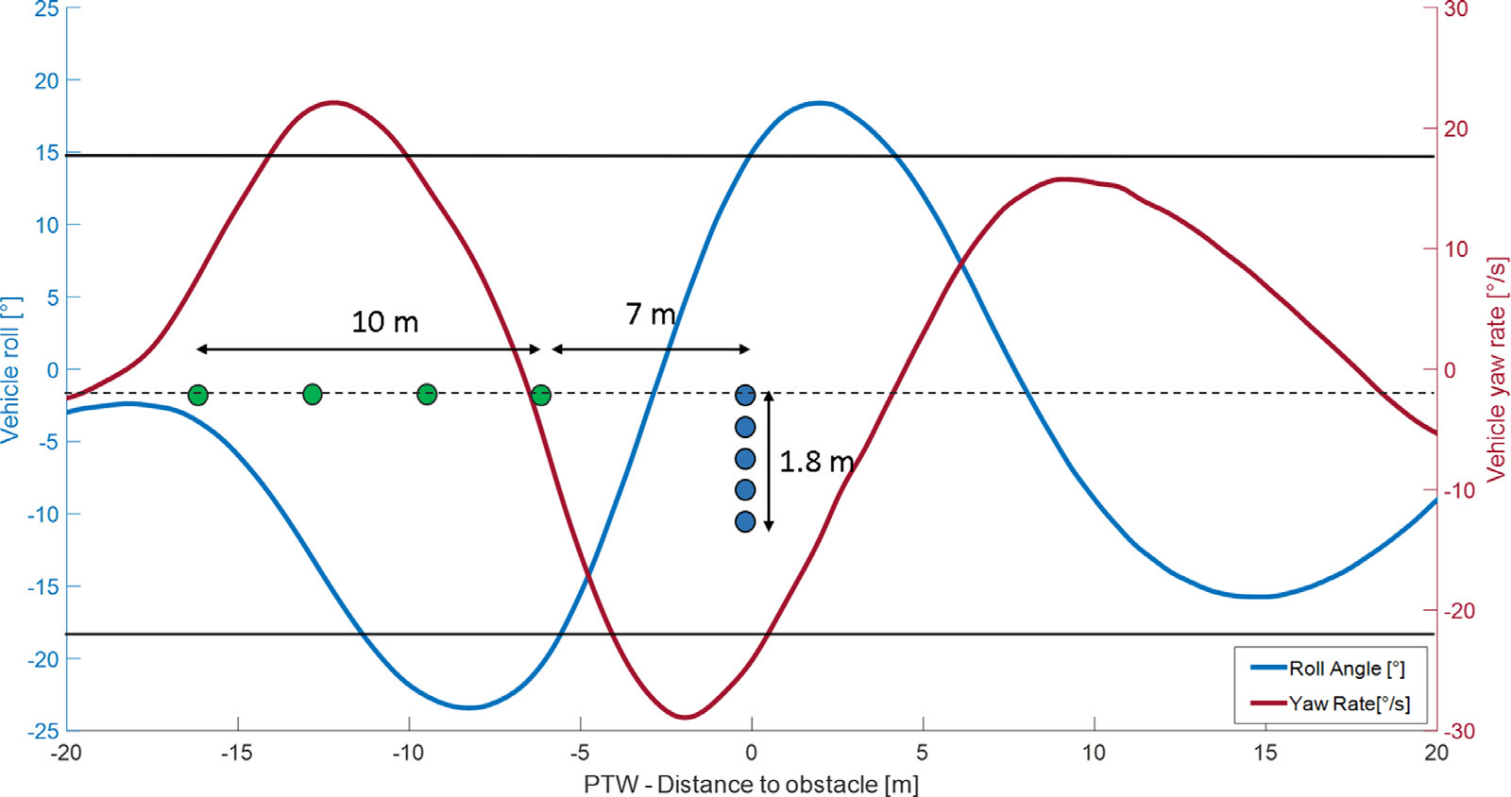
Lane change maneuver.

The lane change maneuver in urban areas can be related to overtaking or swerving, both situations where MAEB is still untested. Even if overtaking is a common behavior among PTWs’ riders, the swerve maneuver is much more relevant in pre-crash situations [7] and therefore it is the maneuver in which MAEB needs to be tested. According to the literature [10], [11], [12], single-lane change was reproduced by placing markers capable of simulating the three phases of the maneuver: i) ‘entry lane’ where the rider drives at a constant speed along a straight path; ii) ‘offset’, a section where the motorcycle is forced to move sideways to change the lane; iii) ‘exit lane’ where the vehicle again follows a straight-line trajectory. As the geometrical parameters of the vehicle (e.g.: wheelbase, trail, caster angle) influence its maneuverability, in this paper both geometrical measures of the lane change section and a set of ranges of the vehicle's dynamic parameters during the maneuver are reported as a reference (see Table 1). The corridor after the maneuver is delimitated to reduce the variability inter-subject and intra-subject during lane change (with and without AB activation) and to position markers orthogonal to the direction of travel in the 'offset' area to simulate an obstacle. In Fig. 1 the geometry of the maneuver and measured vehicle parameters are reported as an example. The AB triggering is performed at the entrance of the transition zone to evaluate its interaction with the execution of the swerve maneuver. In Fig. 3 a picture of a test vehicle during the execution of the maneuver and the set-up of the markers is displayed.

#### Slalom section

**Fig. 2:**
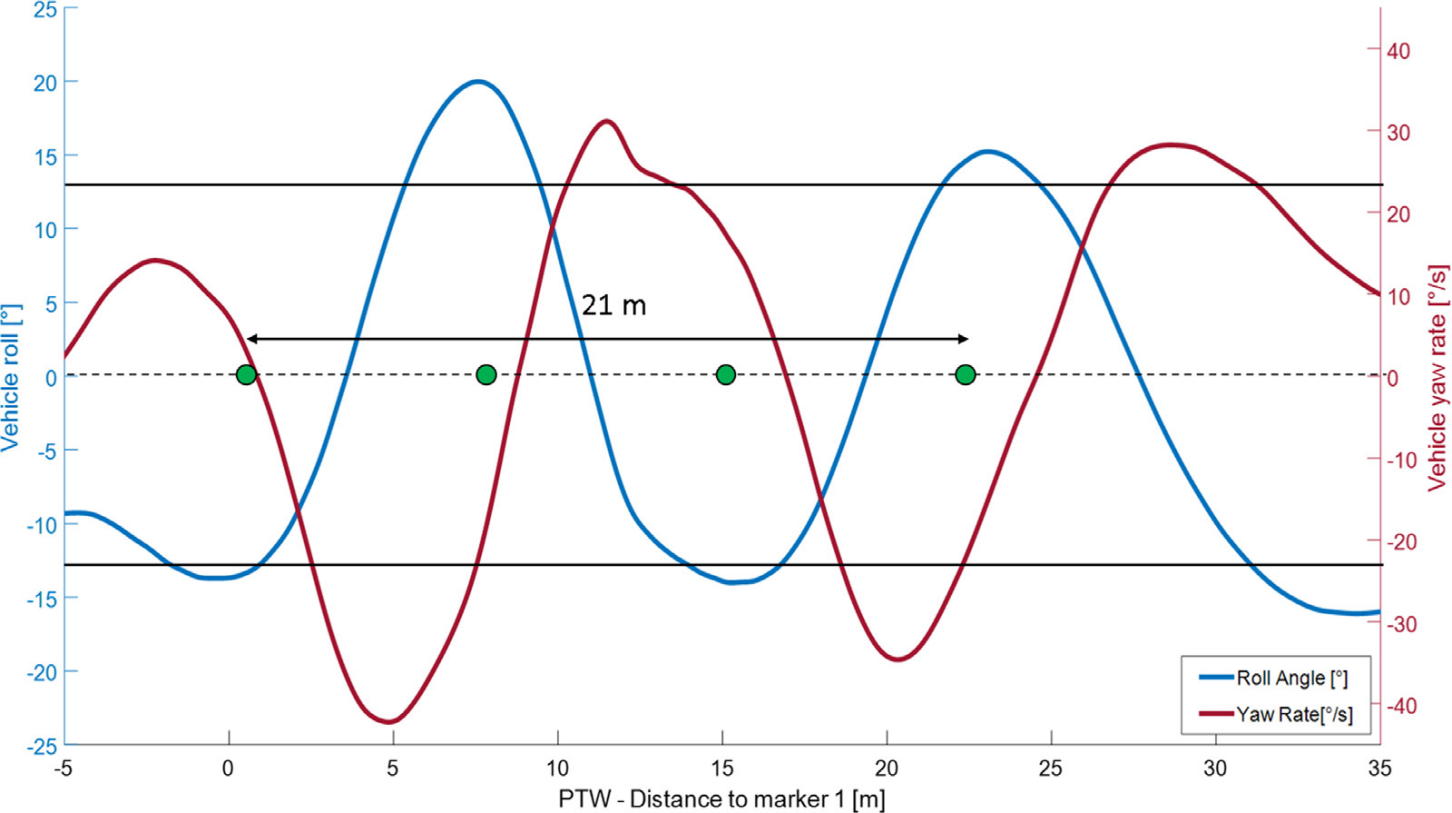
Slalom maneuver.

**Fig. 3:**
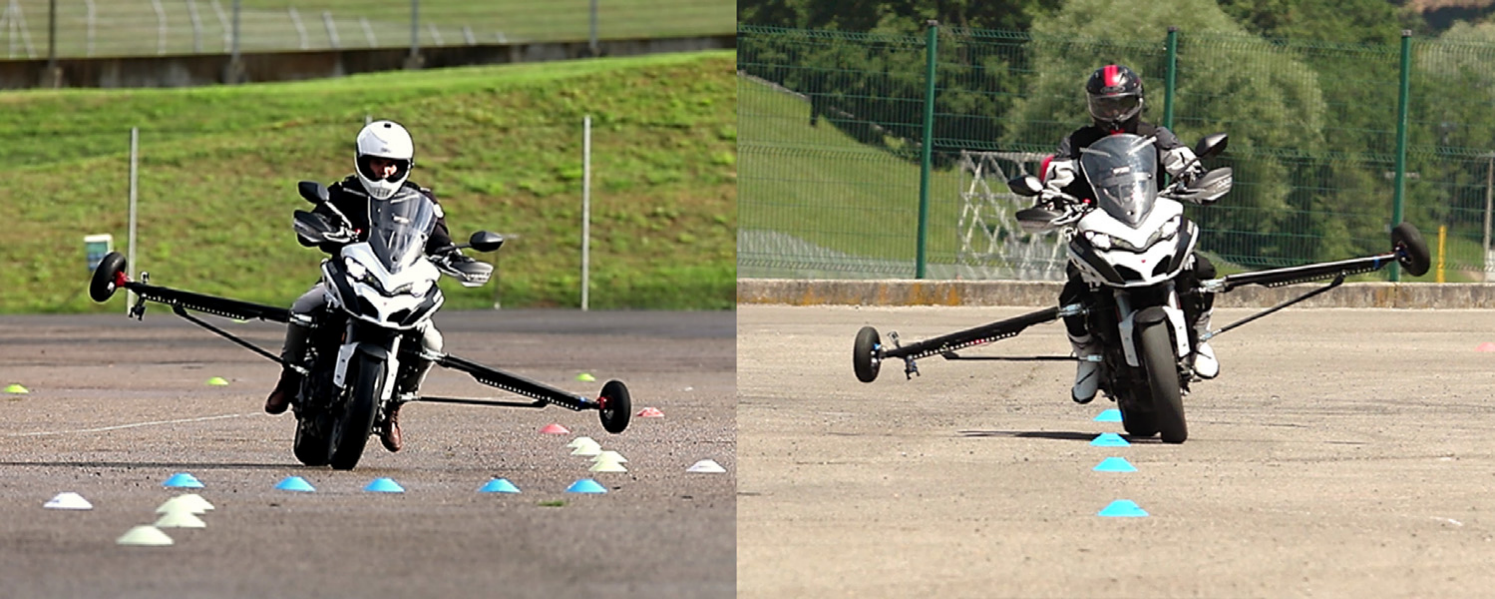
Test vehicle during Lane-change (left) and Slalom (right) maneuvers.

During normal urban riding, it is common to have situations in which the vehicle dynamic is complex, for example during traffic filtering. Although in many states it is declared illegal and in others it is subject to strict regulation [13], traffic filtering is a common behavior among PTW riders to reduce time spent in traffic and to gain the head of queues. Even if these conditions are not those where MAEB could bring clear benefits, it is necessary to test it in this dynamic maneuver, where the rider is moving continuously and therefore less prepared to a reactive action. This is necessary to assess its safety and riders’ reactions (caused by a possible false activation) in such a complex maneuver. In order to reproduce these conditions, a slalom maneuver guided through some markers is included in the test track. Indeed, this path allows reproducing the vehicle's lateral movements and actions on the handlebars made by riders. Slalom is reproduced placing markers with a distance in a range of 7–14 m [10] from each other on a straight line depending on desired travel speed. The distance between markers and the travel speed is selected according to the maneuverability and dynamic behavior of the experimental vehicle (see Table 1). In Fig. 2 the geometry of the maneuver and measured vehicle parameters are reported as an example. In Fig. 3 a picture of a test vehicle during the execution of the maneuver and the set-up of the markers is displayed.

#### Cornering section

A simple way to reproduce a curve is to use markers to define a curved path with a constant radius of curvature. Assuming a steady turning, from the equation of the motorcycle dynamics the necessary radius of curvature can be found according to the target roll and speed to be achieved. To reproduce urban scenarios, the AB is tested in curves with low velocities (25–40 km/h) and small lean angles (20–30°) (see Table 1). Nowadays there are no field experiments with AB activation in curves, although some applications in curves on real accidents have been studied via numerical simulations [14]. However, even if the potential benefits of MAEB in cornering are few and still not clear, it is nevertheless important to study the behavior of riders in the event AB activation during leaning.

Finally, before starting tests with participants both the test vehicle with the AB device and test track are intensely tested to guarantee high safety and reproducibility of the test. The AB device is tested under different environmental (temperature, wind, humidity) and asphalt adherence conditions. Preliminary tests are also useful to assess if the track was correctly designed and if the escape routes were adequate for the implemented maneuvers. Finally, the test protocol is thoroughly tested by several researchers and professional riders, in order to identify critical details of the test such as excessive physical or mental effort.

### Participants recruitment and selection

Before starting with the participant's recruitment, the whole study obtains the approval of an ethical committee, since it is mandatory involving participants in testing active safety system such as MAEB [15,16]. After that, to proceed with participants’ recruitment, a database of potential participants is created collecting information, such as demographics and general opinions on motorcycle safety systems, useful to select a representative sample. This is done through a recruitment questionnaire, which was digitalized and shared with motorcycle clubs, social networks, etc.

The selection phase leads to a sample wide enough to have statistical power and with high representativeness compared to PTW users in urban areas including males and females with different ranges of age. Due to the lack of normative data on which to base an accurate power calculation a convenience sample size of at least 20 participants is used, consistently with the literature examining driving performance [17], driver kinematics [18] or response time [19]. In the previous studies, tests were conducted with professional riders [1,3] and with common riders [2,3]. In order to assess the acceptability of the MAEB for the common users, it is required to continue testing it with this kind of participants. Since with non-professional riders the MAEB assessment could be strongly influenced by the selection of the sample of participants, participants with comparable profiles and riding experience are selected. Due to the limited development of the MAEB, novice riders are excluded and participants with a minimum riding experience are selected (2 years of riding or 10,000 km travelled). Moreover, the selected participants are those who ride weekly a motorcycle comparable to the test vehicle, in order to reduce bias due to different PTW styles.

### Test vehicles

The appropriate test vehicles for these tests are the ones most used in the urban environment (example: moped, scooter and light motorcycle). However, it is easy to presume that the first MAEB systems that will be installed on high-end motorcycles, such as sports style or touring motorcycles. Consequently, such vehicles are also adequate for the experiments. The experimental motorcycle is a standard production vehicle with add-on of minimal non-invasive equipment and instrumentation, to ensure a relaxed and natural ride for the participants during the tests. Moreover, in order to increase the safety of participants during tests, the experimental PTW is equipped with standard ABS or better with Motorcycle Stability Control (MSC) and Traction Control (TC), since it is presumed that MAEB will be implemented on PTWs equipped with these systems.

### Data collection and instrumentation

To measure the effect of AB interventions on riding stability, different procedures can be applied, which can be classified into performance, physiological and subjective measures. They differ in their degree of effectiveness and reliability and the resources required for their implementation [20].

#### Performance measures

The effect of the AB interventions is assessed measuring the performance in the riding tasks or maneuvers. Standard performance measures are the Mean Deviation (MD) from a nominal model, or the MD from a participant's baseline [20]. Additionally, kinematic thresholds defined in previous studies as reference for vehicles [21,22] and rider [23,24] are applied to measure the controllability and the stability of the vehicle during the maneuvers.

##### Vehicle data

Vehicle 3-axis accelerations and 3-axis gyro, brake pressures, throttle and steering position, clutch usage, speed and AB diagnostics signals are the basic variables to monitor if the response of the system corresponds to the designed one and to assess the riding behavior. To assess the performance of the system, the longitudinal acceleration is measured in two different ways to have a backup measure.

##### Rider position and kinematic data

The movement of the rider's body is registered with an Inertial Measurement Unit to investigate the interaction between the pilot and the vehicle during the activation of the system. The main variables collected are angle hip-chest, position (attitude), velocity and accelerations (linear and angular) of the rider's chest. Body position and movements are also recorded by video-cameras using anatomical landmarks of interest on segments of the body. An extra camera on a tripod out of the circuit records the maneuvers for qualitative analysis.

#### Physiological measures

The galvanic skin response (GSR) and the electromyography (EMG) may be employed to assess how demanding is for the rider to maintain the control of the vehicle [5]. Temperature and heart rate are also used to assess the responses of the body with physiological indicators [2].

#### Subjective measures/self-report measures

**Fig. 4:**
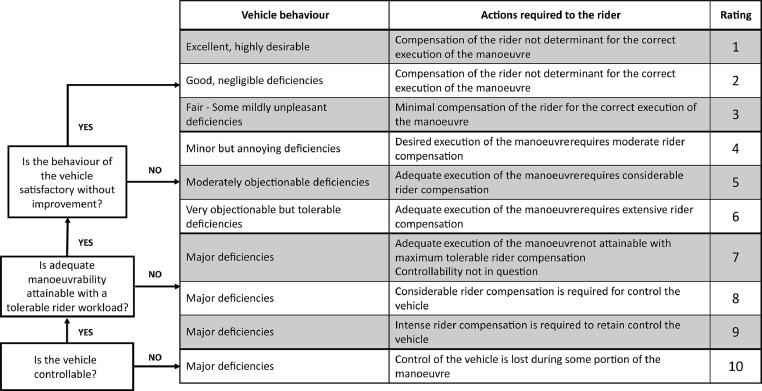
PTW-adjusted Cooper-Harper rating scale.

Subjective measures with questionnaires are a good solution to obtain additional data of interest related to the perception of participants. By means of clear and concise questions, questionnaires gather information related to the demands imposed on the subject (mental, physical, and temporal demands) and information related to the perception of the AB interventions and the effort required to control the PTW (compensate the AB action). To evaluate the physical and mental effort the Borg scale [25] is used, since it was widely adopted as an indicator to monitor exercise intensity. In order to have a comparable valuation of the controllability of the system, an adapted Cooper-Harper rating scale is employed [26,27]. This scale, which is a rating scale that is widely adopted to assess the controllability of aircrafts, was adjusted to assess the controllability of a PTW. As reported in Fig. 4, the rating in the scale is from one to ten, where one means an excellent behavior of the vehicle (the rider is not required to compensate the intervention of the system to maintain the desired trajectory), and ten means a loss of control. Additionally, a brief interview after the tests provides extra information about the participant's impressions.

## Test protocol specifications

The test protocol starts with a brief explanation of the test vehicle and its special controls. A short warm-up is also required to enable the participant to be familiar with the vehicle and the track before testing the intervention of AB. To evaluate participant control skills, the participants are asked to perform a set of manual hard braking until stopping the vehicle [5]. The measures of the response of the rider during the manual braking were useful to be compared with those taken during the AB interventions.

Since grating participants’ safety is the main concern and an ethical duty in research involving human beings [15,16], the design of the experiment in the approach of the participant to the intervention of AB must find a compromise between the optimal design [20] and safety. Therefore, before testing an unexpected intervention of AB the participant experiences expected interventions with levels of deceleration considered safe in the literature (at present, up to 2 m/s^2^ [2,3]). Even if it could be a source of bias due to learning effects, this lets the participant have a safe familiarization with AB before testing new decelerations and maneuvers. Furthermore, measures of these declared AB interventions can be used to compare the rider's response with those responses during the unexpected interventions of the test.

Activations of the system in unexpected conditions are anticipated by an explanation of the investigator, who introduces the intervention in terms of deceleration, jerk and maneuvers with possible system activation. The information is necessary to allow the rider to exclude from the trial activation of AB any of the maneuvers, if reputed not adequate to her/his riding level. Afterwards, the activations of the system in unexpected conditions are tested, triggering the AB in the different maneuvers according to a pseudorandom time scheme and starting with the lowest level of deceleration and jerk. At the end of each trial, the investigator asks some feedback from the participant before to proceed to test higher levels of deceleration or jerk.

The overall duration of the test is no longer than two hours, in order to avoid excessive fatigue and psychological overload of the participant. This time also includes some breaks to let the participant rest between different riding sessions.

The guidelines and principles proposed in this section were applied as an example in the test protocol contained in Table 2. The protocol is divided into different parts, one for each test phase; the AB test sessions can be repeated for each level of deceleration or jerk included in the test.

**Table 2:** Example of the test protocol.

Test phase	Description	Duration [min]
**Informing Participant**	The participant receives all the information about the test, all the forms needed to take part in the test being checked by the researcher. After inspecting all the protective equipment and showing to the participant the test track, the researcher shows the test vehicle and its special commands. A demonstration of riding in the test track, performed by a team member, can be included to show how to approach the track and the maneuvers.	10
**Familiarization**	The participant has a limited time to warm-up and becomes familiar with the test vehicle, the track and all the maneuvers that are included in the test.	10–15
**Base-line braking measures**	The participant performs, at an initial speed of 40–45 km/h, manual stop maneuvers braking at three different levels of deceleration: corresponding approximately to the 30%, 50% and 90% of the maximum deceleration that he/she can achieve with the test vehicle.	10
**AB Familiarization**	The participant tests declared activation of the AB system at the different levels of deceleration planned to be tested in straight-line at a speed of 40–50 km/h.	5
**First AB test session**	The participant tests the unexpected activations of the system at the lowest level of deceleration. The interventions are defined at different points of the track in a pseudorandom way along the defined number of laps to reduce predictability. The researcher explains the maneuvers and where the system can be activated and asks the rider whether he/she wants to exclude in the trial the activation in any of the maneuvers. Eventually, the participant may ask to test one declared activation to include/exclude any of the maneuvers before the session with unexpected activations.	Number of Activations x 100 s
**Break**	The participant has some time to rest and completes a brief questionnaire about demands related to interaction with the system.	10
**Further AB test sessions**	The participant tests the activation of the system with higher levels of deceleration or jerk, following the same procedure of the first session. Again, the participant may ask to test one declared activation to include/exclude any of the maneuvers. The session ends with a break and a brief questionnaire.	Number of Activations x 100 s
**Overall Questionnaire**	The participant fills in a final questionnaire about the entire test and the MAEB system. A recorded interview can also be included.	15

## Test method validation

The procedure and the design criteria presented in this paper were employed to define a test protocol which was applied to further investigate the feasibility of MAEB [28,29]. Field tests were conducted involving 55 participants testing Automatic Braking intervention on three different PTWs. Field tests executed with the procedure presented in this paper were carried out [28,29]. The results coming from the analysis of these tests will provide a comprehensive understanding of the feasibility and the acceptability of the Autonomous Emergency Braking system applied to PTWs. In this section results from the final questionnaire filled-in by participants concerning the execution of the test are displayed, in order to validate the methods proposed in this paper.

A first validation of the proposed test procedure is that all the 55 participants completed the full test protocol and no potentially dangerous situations occurred for them nor the research team. This test campaign allowed to test the effects of automatic braking events deployed in different maneuvers and riding condition and different AB working parameters.

**Fig. 5:**
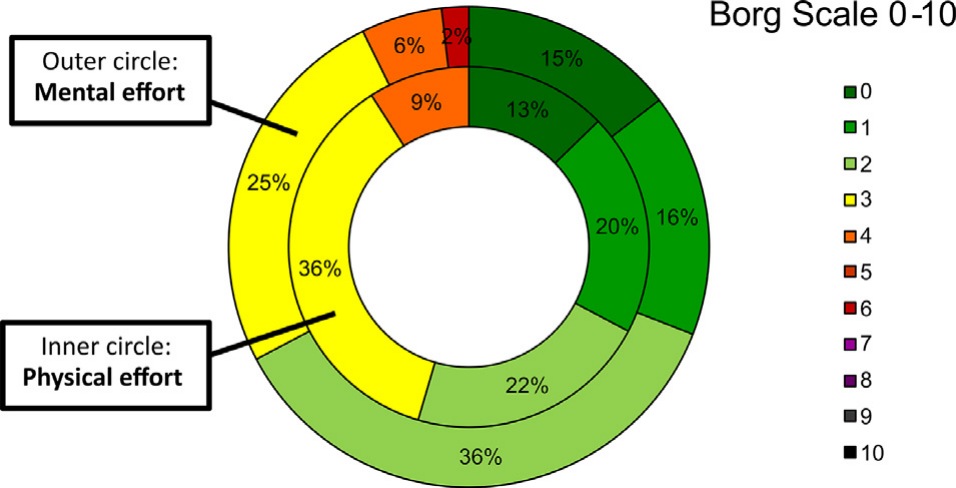
Participants’ rating of Physical and Mental effort - Borg scale (0= No effort – 10=Maximal effort).

Fig. 5 shows the distribution of the participants’ rating regarding the mental and the physical effort required to complete the test. The complete test was considered generally easy manageable from both mental and physical point of view. Employing the Borg rating scale, participants gave an average score of 2.1 (SD =1.2) to the physical effort and 2.0 (SD = 1.3) to the mental effort, both scores represent a light effort required to the participants to perform the test.

**Fig. 6:**
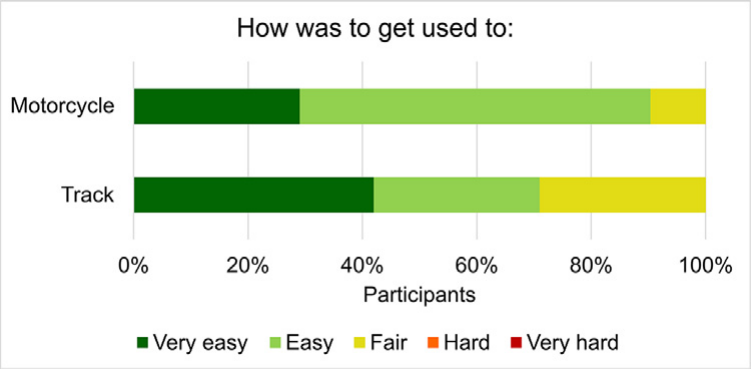
Participants’ rating of the warm-up phase.

Concerning the warm-up test phase, all the participants rated the time given for the familiarization with the test vehicle and the track as enough (49%) or abundant (47% abundant, 4% very abundant). In Fig. 6 is displayed the participants’ rating of the warm-up test phase session. For 90% and 70% of participants, becoming familiar with respectively the motorcycle and the test track with the included maneuvers, turn out to be easy or very easy. Nevertheless, no excessive strains to obtain an acceptable familiarization were reported by the remaining participants.

## Planned data analysis

Field testing the intervention of an active safety system such as MAEB is a mandatory process before its introduction on standard vehicles (see for example what was done for AEB [30,31]. This is because it is required to prove its real feasibility and acceptability among end-users, ensuring MAEB safety in real-world scenarios. Therefore, after testing the intervention of MAEB following the guidelines and principles proposed in previous paragraphs, accurate data analysis is required to prove its acceptability and feasibility. The questionnaires and the feedback obtained from the participants during the final interview provide important hints on the acceptability of the systems for end-users and its working parameters. From questionnaires, information can be also acquired about the execution of the test and the tested AB device reproducing the effects of the MAEB system. The riders’ movements are analyzed to investigate the effects of MAEB, monitoring their body reactions during the different phases of the automatic decelerations. This information is compared with data from questionnaires and with the movements of the body during normal riding and manual braking. Data from the vehicle is analyzed to have a complete characterization of the intervention of MAEB and the behavior of the riders during its interventions, focusing on PTW controls (throttle, clutch, brakes, steering angle) and PTW stability parameters. This analysis verifies or questions the subjective results coming from questionnaires with objective data. The data analysis also highlights if the intervention of MAEB can cause some hazards for the rider or reduce his/her ability to maneuver in pre-crash situations. These can be done using the fall detection models available in the literature for PTWs [21], [22], [23],32].

## Conclusion

This paper describes a procedure to field test the intervention of an active safety system for motorcycles as MAEB (Motorcycle Autonomous Emergency Braking). Field test specifications concerning testing conditions, maneuvers, participants requirements, vehicles and instrumentation requirements are provided in order to support its design and robust execution. Lastly, an outline of data analysis is included.

The proposed guidelines and principles provide complete support to design a test protocol to further investigate the intervention and the feasibility of advanced assistance systems for motorcycles such as MAEB. Furthermore, this approach can be used as a reference for designing a field test for other active safety systems for PTW.

## Additional information

### Background of motorcycle autonomous emergency braking testing

A powered two-wheeler (PTW) colliding a stationary or slowly moving vehicle was found to be a common crash scenario in developed traffic contexts such as Europe and Australia [33,34]. In these situations, an automated braking response of the motorcycle was indicated as a plausible solution to reduce injury outcomes for the riders [35], assuming that such intervention should be deployed when the collision has become inevitable.

Researchers have considered whether common riders may be able to handle their vehicle under automated braking and what are the parameters of such intervention. The first documented experiment was conducted in the lab using a backward accelerating sledge, putting effort to produce unexpected events with equivalent decelerations of up to 3 m/s^2^ [24]. The first on-road tests were conducted involving professional riders approaching a target obstacle with a test PTW equipped with a laser-scanner and producing automatic decelerations up to 7 m/s^2^. A limitation of such tests was that participants were not presented with genuine unexpected events [1,36]. In an attempt to reduce the level of predictability of the automated braking events, the following experiment was conducted without obstacles and involving common user participants. The so-called “Wizard of Oz” approach was in place, in which the investigator activated the automated decelerations (up to 2 m/s^2^) of the test vehicle via remote control [2]. New experiments were then conducted with professional riders testing undeclared automatic braking events at speeds up to 80 km/h, with decelerations up to 7 m/s^2^ and jerk up to 12 m/s^3^ [3]. These tests involved a moving target obstacle (a car mock-up trailer) reproducing a medium speed car-following test scenario. This study suggested that automatic decelerations greater than 3 m/s^2^ can be controlled by common riders in straight-line motion.

In summary, previous research findings based on activation along a straight trajectory support the autonomous emergency braking for motorcycles (MAEB), but new tests are required to assess the feasibility of MAEB interventions for common riders when adopting decelerations greater than 2 m/s^2^.

As regards the intensity of the automatic braking event, early studies that analyzed the effectiveness of MAEB suggested that decelerations of 3 m/s^2^ may not be sufficient to reduce the likelihood of sustaining serious injuries [37]. If confirmed, the feasibility of higher decelerations becomes a critical factor for future development and implementation of MAEB.

As concerns the intervention scenarios to be considered, the activation in straight-line motion is certainly important, since in-depth crash investigations showed that in most pre-crash phases the motorcycle is not in a turn. However, in typical riding conditions, including pre-crash conditions where MAEB may apply, the motorcycle moves along trajectories that involve tilting oscillations (see for example [37]). Also, a lateral avoidance maneuver is often attempted by the rider during the pre-crash phase [7]. For these reasons, test scenarios other than the simple straight-line motion are warranted for a better understanding of the possible risks and possible applications of MAEB deployment before a crash in the real world.

### Process employed to define the proposed test method

The guidelines and design criteria proposed in this paper were obtained through two linked phases: a literature review and a pilot test. First, to determine more realistic intervention setting for MAEB and new working parameters to be tested, a comprehensive literature review of the previous studies on MAEB was carried out. Researches concerning field testing were analyzed to find out which working parameters of MAEB were tested and recommended by literature so far, and which scenarios and maneuvers were already tested and considered safe. Works on benefits assessment and simulations were also analyzed to identify which conditions of intervention are more relevant or critical for MAEB. Moreover, in order to define new realistic intervention settings, a literature review of previous studies concerning PTWs maneuvers definition and testing was carried out.

After the literature review, using a vehicle provided with an Automatic Braking (AB) device previously developed by authors [38], a series of intensive pilot testing was conducted involving four researchers and expert riders. Tests included riding conditions relevant for MAEB activation in a real-world setting. The riders were aware of the test scope, but they performed an objective judgement on the activation for defining conditions viable for participants with any expertise level and unaware of the activation timing of the system. Specific AB activation parameters were defined for each tested maneuver. Finally, a test protocol based on guidelines coming from both literature review and field testing was defined and intensively tested in following sessions.

## Declaration of Competing Interest

The authors declare that they have no known competing financial interests or personal relationships that could have appeared to influence the work reported in this paper.

## References

[1] Savino G., Pierini M., Baldanzini N. (2012). Decision logic of an active braking system for powered two wheelers. J. Automob. Eng..

[2] Savino G., Pierini M., Thompson J., Fitzharris M., Lenné M.G. (2016). Exploratory field trial of motorcycle autonomous emergency braking (MAEB): considerations on the acceptability of unexpected automatic decelerations. Traffic Inj. Prev..

[3] Merkel N., Pless R., Scheid K., Winner H. (2018). Limits of autonomous emergency brake systems for powered two-wheelers – an expert study. Proceedings of the 12th International Motorcycle Conference.

[4] Savino G., Giovannini F., Fitzharris M., Pierini M. (2016). inevitable collision states for motorcycle-to-car collision scenarios. Proceedings of the IEEE Transactions on Intelligent Transportation Systems.

[5] Huertas-Leyva P., Nugent M., Savino G., Pierini M., Baldanzini N., Rosalie S. (2019). Emergency braking performance of motorcycle riders: skill identification in a real-life perception-action task designed for training purposes. Transp. Res. F Traff. Psychol. Behav..

[6] Huertas-Leyva P., Baldanzini N., Savino G., Pierini M. (2021). Human error in motorcycle crashes: a methodology based on in-depth data to identify the skills needed and support training interventions for safe riding. Traffic Inj. Prev..

[7] Penumaka A.P., Savino G., Baldanzini N., Pierini M. (2014). In-depth investigations of PTW-car accidents caused by human errors. Saf. Sci..

[8] Lee T.C., Polak J.W., Bell M.G.H. (2009). New approach to modeling mixed traffic containing motorcycles in urban areas. Transp. Res. Rec..

[9] Anderson R., Doecke S., Mackenzie J., Ponte G. (2013). Potential benefits of autonomous emergency braking based on in-depth crash reconstruction and simulation. Proceedings of the 23rd International Technical Conference on the Enhanced Safety of Vehicles.

[10] Cossalter V., Lot R., Rota S. (2010). Objective and subjective evaluation of an advanced motorcycle riding simulator. Eur. Transp. Res. Rev..

[11] Cossalter V., Sadauckas J. (2006). Elaboration and quantitative assessment of manoeuvrability for motorcycle lane change. Veh. Syst. Dyn..

[12] Cheli F., Pezzola M., Taroni N., Mazzoleni P., Zappa E. (2011). Driver's movements influence on the lateral dynamic of a sport motorbike. Proceedings of the 19th Mediterranean Conference on Control & Automation (MED 2011).

[13] T. Rice, L. Troszak, T. Erhardt, Motorcycle lane-splitting and safety in California, 2015.

[14] Savino G., Giovannini F., Piantini S., Baldanzini N., Pierini M. (2015). Autonomous emergency braking for cornering motorcycle. Proceedings of the 24th ESV Conference.

[15] Concil of Europe (1997). Convention on human rights and biomedicine. Eur. Treaty Ser..

[16] National Health, Medical Research Council (2007). Australian Research Council, Australian Vice -Chancellors. Committee, National Statement on Ethical Conduct in Human Research.

[17] Duan J., Li R., Hou L., Wang W., Li G., Li S.E., Cheng B., Gao H. (2017). Driver braking behavior analysis to improve autonomous emergency braking systems in typical Chinese vehicle-bicycle conflicts. Accid. Anal. Prev..

[18] Osth J., Olafsdóttir J.M., Davidsson J., Brolin K. (2013). Driver kinematic and muscle responses in braking events with standard and reversible pre-tensioned restraints: validation data for human models. SAE Tech. Pap..

[19] Aust M.L., Engström J., Viström M. (2013). Effects of forward collision warning and repeated event exposure on emergency braking. Transp. Res. F Traff. Psychol. Behav..

[20] Cozby P., Bates S. (2015). Methods in Behavioral Research. https://www.mheducation.com/highered/product/methods-behavioral-research-cozby-bates/M9781259676987.html.

[21] Boubezoul A., Espié S., Larnaudie B., Bouaziz S. (2013). A simple fall detection algorithm for powered two wheelers. Control Eng. Pract..

[22] Huertas-Leyva P., Savino G., Baldanzini N., Pierini M. (2020). Loss of control prediction for motorcycles during emergency braking maneuvers using a supervised learning algorithm. Appl. Sci..

[23] Bellati A., Cossalter V., Lot R., Ambrogi A. (2006). Preliminary investigation on the dynamics of motorcycle fall behavior: influence of a simple airbag jacket system on rider safety. Proceedings of the 6th International Motorcycle Conference.

[24] Symeonidis I., Kavadarli G., Erich S., Graw M., Peldschus S. (2012). Analysis of the stability of PTW riders in autonomous braking scenarios. Accid. Anal. Prev..

[25] Borg G. (1998). Borg's Perceived Exertion and Pain Scales.

[26] G.E. Cooper, R.P. Harper, The use of pilot rating in the evaluation of aircraft handling qualities, 1969. https://ntrs.nasa.gov/archive/nasa/casi.ntrs.nasa.gov/19690013177.pdf.

[27] Savino G., Pierini M., Lenné M.G. (2016). Development of a low-cost motorcycle riding simulator for emergency scenarios involving swerving. Proc. Inst. Mech. Eng. D J. Automob. Eng..

[28] Lucci C., Huertas-Leyva P., Marra M., Pierini M., Savino G., Baldanzini N. (2020). Autonomous emergency braking system for powered-two-wheelers: testing end-user acceptability of unexpected automated braking events deployed in typical pre-crash trajectories. Proceedings of the 13th International Motorcycle Conference.

[29] Marra M., Lucci C., Huertas-Leyva P., Baldanzini N., Pierini M., Savino G. (2020). The future of the Autonomous emergency braking for powered-two-wheelers : field testing end-users’ acceptability in realistic riding manoeuvres. Proceedings of the IOP Conference Series Materials Science and Engineering.

[30] Fecher N., Hoffmann J., Winner H., Fuchs K., Abendroth B., Bruder R. (2008). Analysis of driver behaviour in autonomous emergency hazard braking situations. Proceedings of the FISITA World Automotive Congress.

[31] Kobiela F., Engeln A. (2010). Autonoumus emergency braking - studies on driver behaviour. ATZ Autotechnol..

[32] Giovannini F., Baldanzini N., Pierini M. (2014). Development of a fall detection algorithm for powered two wheelers application. SAE Tech. Pap..

[33] Savino G., Rizzi M., Brown J., Piantini S., Meredith L., Albanese B., Pierini M., Fitzharris M. (2014). Further development of motorcycle autonomous emergency braking (MAEB), what can in-depth studies tell us? A multinational study. Traff. Inj. Prev..

[34] Savino G., Mackenzie J., Allen T., Baldock M., Brown J., Fitzharris M. (2016). A robust estimation of the effects of motorcycle autonomous emergency braking (MAEB) based on in-depth crashes in Australia. Traff. Inj. Prev..

[35] Savino G., Lot R., Massaro M., Rizzi M., Symeonidis I., Will S., Brown J. (2020). Active safety systems for powered two-wheelers : a systematic review. Traff. Inj. Prev..

[36] Giovannini F., Savino G., Pierini M., Baldanzini N. (2013). Analysis of the minimum swerving distance for the development of a motorcycle autonomous braking system. Accid. Anal. Prev..

[37] Gil G., Savino G., Piantini S., Pierini M. (2018). Is stereo vision a suitable remote sensing approach for motorcycle safety? An analysis of LIDAR, RADAR, and machine vision technologies subjected to the dynamics of a tilting vehicle. Proceedings of the 7th Transport Research Arena.

[38] Lucci C., Berzi L., Baldanzini N., Savino G. (2019). Remote controlled braking actuation for motorcycle safety system development. Proceedings of the IEEE 5th International Forum on Research and Technology for Society and Industry.

